# Retro-MoRFs: Identifying Protein Binding Sites by Normal and Reverse Alignment and Intrinsic Disorder Prediction

**DOI:** 10.3390/ijms11103725

**Published:** 2010-09-29

**Authors:** Bin Xue, A. Keith Dunker, Vladimir N. Uversky

**Affiliations:** 1 Center for Computational Biology and Bioinformatics, Indiana University School of Medicine, Indianapolis, IN 46202, USA; E-Mails: binxue@iupui.edu (B.X.); kedunker@iupui.edu (A.K.D.); 2 Institute for Intrinsically Disordered Protein Research, Indiana University School of Medicine, Indianapolis, IN 46202, USA; 3 Department of Molecular Medicine, University of South Florida, Tampa, FL 33612, USA; 4 Institute for Biological Instrumentation, Russian Academy of Sciences, 142290 Pushchino, Moscow Region, Russia

**Keywords:** reverse, retro, invert, alignment, intrinsic disorder, PONDR-RIBS

## Abstract

Many cell functions in all living organisms rely on protein-based molecular recognition involving disorder-to-order transitions upon binding by molecular recognition features (MoRFs). A well accepted computational tool for identifying likely protein-protein interactions is sequence alignment. In this paper, we propose the combination of sequence alignment and disorder prediction as a tool to improve the confidence of identifying MoRF-based protein-protein interactions. The method of reverse sequence alignment is also rationalized here as a novel approach for finding additional interaction regions, leading to the concept of a retro-MoRF, which has the reversed sequence of an identified MoRF. The set of retro-MoRF binding partners likely overlap the partner-sets of the originally identified MoRFs. The high abundance of MoRF-containing intrinsically disordered proteins in nature suggests the possibility that the number of retro-MoRFs could likewise be very high. This hypothesis provides new grounds for exploring the mysteries of protein-protein interaction networks at the genome level.

## 1. Introduction

All the challenges in biological research may come down to the molecular level and be conquered by various physical and chemical interactions among various bio-molecules, such as proteins, DNA, and RNA. The peculiarities of the interaction patterns between these molecules can be represented as various interaction networks. The sequential and spatial effects of these interaction networks control the origin, development, and evolution of all living organisms. Therefore, identifying possible interactions among various bio-molecules is of great importance for current biological science.

However, identifying inter-molecular interactions, especially *in vivo*, is not a trivial task. Traditional experimental methods are both time- and cost-consuming. Advanced experimental methods, such as yeast 2 hybrid, although fast and efficient, have significant false positive and false negative rates. In spite of these experimental difficulties, developments in bioinformatics have made a great contribution in this area. Techniques based on sequence alignment become the most basic but very powerful tools for identifying structures, functions, and mutual interactions among bio-molecules. The underlying principle supporting the application of sequence alignment for studies of protein structure and function is the well-accepted sequence-structure-function paradigm: Sequence determines the structure; Structure determines the function. Here, for the simplicity of description, function can be interpreted as the ability of a bio-molecule (protein) to interact with another molecule. Apparently, this ability is due to the local structure determined by the spatial arrangement of amino acids, which is encoded in protein’s amino acid sequence. If two proteins have very similar sequences, their structures are very likely to be highly similar, and, therefore, they may have a very similar interaction mode with the third molecule. This is the underlying basis for the sequence alignment.

Although the underlying principles of sequence alignment have integrated many atomic details implicitly, the application of sequence alignment raised itself to the phenomenon level. In fact, only the sequential order of the symbols of amino acid residues is required for the alignment: If the sequential orders of amino acids in two proteins are similar to each other, their structures and functions are likely to be similar to each other as well. This logic can also be applied to protein domains or segments: if two segments have similar sequential order of amino acids, they may form similar local 3D structures and perform similar functions. Many conserved motifs and active sites are identified through this process. Although for highly conserved segments, the structural and functional prediction from sequence alignment is highly convincing, there are a lot of uncertainties for moderately similar segments. Furthermore, there is another intriguing question: if the segment A is reversely identical to the segment B, are they similar in structure and function? This is a very important question. In fact, if the answer to this question is positive or partially positive under some conditions, the interaction patterns ascribed to bio-molecular pathways and networks could be far more complicated.

Several pioneer studies have been performed to address the issue of proteins with reversed sequences (retro-proteins). As early as 1992, Schoniger and Waterman introduced inversion (reversed complements) into sequence alignment [1]. However, it was not clear whether the reversed sequences have the same structure and function as the original, normal-order, sequences. Later, studies of different proteins and models produced contradictory results. For example, computational simulations based on the coarse grained model of the domain B of staphylococcal protein A indicated that the retro-sequence would likely have a structure similar to that of the original sequence [2]. However, according to the full-atom simulations and experiments, this retro-protein was shown to be essentially unfolded [3]. In addition, retro-proteins produced from sequences of SH3 domain and B1 domain of Streptococcal protein G were also shown to be unfolded by both full-atom simulation and experiments [3]. Several reversed polypeptides possessed conformations totally different from their conformations of original sequences [4,5]. Apparently, the results of the coarse grained modeling of the retro-proteins were different from all-atom models. Very likely, the difference comes from the differences of the local, detailed structures of amino acids; *i.e.*, dihedral angles and side chain orientations, which are omitted in the coarse grained model. In addition to these individual examples, a statistical study revealed that retro-sequences are unlikely to have fold similar to the original sequences [6,7]. In contrast to these various studies, there are still other experimental examples showing that although retro-sequences may have not only a stable 3D structure [8], but may also have functions similar to original sequences [9–12].

All the above mentioned studies were focused on ordered proteins with unique rigid structures. These studies clearly showed that the folding of retro-proteins is generally perturbed compared to their normal-sequence counterparts and that the presence of reversed segments may dramatically change the rigid 3D structure of an ordered protein. However, what happens with respect to reversed sequences of intrinsically disordered proteins (IDPs)? IDPs do not have unique 3D structures under physiological conditions. Instead, they form an ensemble of flexible conformations. Many crucial biological functions are carried out by these dynamic and flexible conformations via structural changes and conformational transformations [13–16]. Although the mentioned conformational flexibility makes IDPs totally different from structured proteins, no study on reversed alignments has been reported for these proteins that lack stable structure.

Since IDPs have much more structural flexibility, the underlying assumptions for sequence alignment of IDPs may be very different from those proposed for aligning the structured proteins. In fact, for structured proteins, when the sequence identity is over 20–30%, the corresponding structural similarity is potentially very high (in terms of RMSD, the difference between two structures of this sequence identity is often less than 2 Å) [17]. How similar are structures and functions of two disordered proteins when their sequence similarity is only 20–30%? How different are structures and functions of IDPs, with normal order and reversed sequences? In other words, how are the structures and functions of IDPs affected by inversion of their amino acid sequences? These questions can hardly be neglected due to the high abundance of IDPs in nature. Typically, 7–30% prokaryotic proteins contain long disordered regions of more than 30 consecutive residues, whereas in eukaryotes the amount of such proteins reaches 45–50% [18–22]. Even in the PDB database, which is highly biased towards structured proteins, ~70% of proteins have regions of missing electron density, *i.e.*, disordered regions [23]. Of these, over 10% have long segments of missing electron density consisting of at least 30 amino acids [24]. As far as the protein functions are concerned, as emphasized by previous studies, 238 out of 710 Swiss-Prot functional keywords were strongly positively correlated with intrinsic disorder, while 302 other functional key words were strongly negatively associated with intrinsic disorder [25].

In this paper, we describe an integrated analysis of the relation between the reversed sequence, sequence alignment, and intrinsic disorder. Furthermore, by combining these three features, we developed a novel protocol for identification of potential protein-protein interaction sites, herein called retro-MoRFs, which are reversed-sequence molecular recognition features. Just as commonly observed for normal-sequence MoRFs, retro-MoRFs are short segments that are expected to have a high propensity for folding-upon-binding and that are located within regions of disorder.

## 2. Results and Discussion

### 2.1. Functional Roles of MoRF Regions in Three Proteins

**Figure 1:**
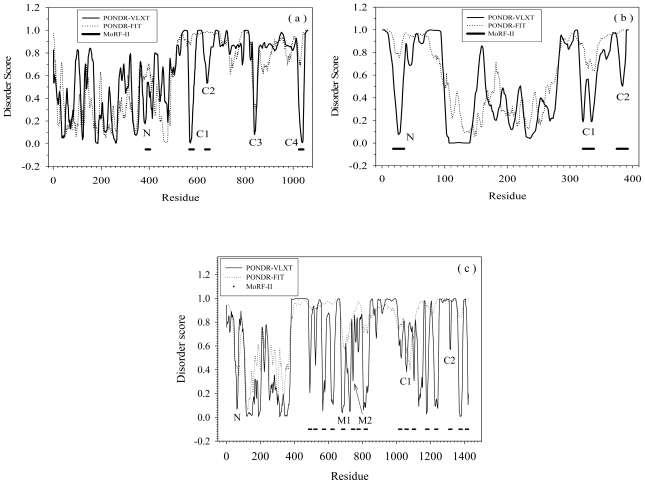
Disorder and MoRF prediction for (**a**) RNase E, (**b**) p53, and **(c)** SRC-3. The thin solid lines are prediction of PONDR-VLXT, dotted lines are prediction from PONDR-FIT, and horizontal bold lines are the MoRF regions identified by MoRF-II predictor. In subset (a), N and C1–C4 correspond to one N-terminal dip and four C-terminal dips of RNase E. In subset (b), N, C1, and C2 stand for N-terminal dip and two C-terminal dips for p53. In (c), there are one N-terminal dip N, two middle-region dips M1 and M2, and two C-terminal dips C1 and C2.

Figure 1 shows disorder prediction and its relation to MoRFs [22,26] for three illustrative proteins: (a) RNase E, (b) p53, and (c) SRC-3. PONDR-VLXT and PONDR-FIT were used to make the disorder predictions. The former predictor is the more sensitive to the local amino acid composition, while the latter is one of the most accurate disorder predictors. As shown by Figure 1(a)–(c), although the general trends are similar, PONDR-VLXT has more intense fluctuation of disordered scores, whereas PONDR-FIT shows smaller fluctuations and more gradual variation overall. The dips reflected by the disorder score fluctuations may correspond to MoRF regions. The protein segments corresponding to these dips have much higher content of hydrophobic residues than their flanking regions. Since increased hydrophobicity favors structure over disorder in PONDR-VLXT, such segments are predicted to be locally structured by this predictor, while the neighboring regions are more strongly predicted to be disordered. From a biological viewpoint, due to high local content of hydrophobic residues, the protein segments corresponding to these dips may have crucial roles in molecular recognition and signaling. However, not all of the dips can be identified as α-MoRFs. In fact, the application of the α-MoRF identifier [22,26] reveal only four α-MoRFs in RNase E (Figure 1(a)), three α-MoRFs in p53 (Figure 1(b)), and sixteen α-MoRFs in SRC-3 (Figure 1(c)).

All the α-MoRFs in Figure 1(a) and (b) were selected for further study. But only four out of sixteen α-MoRFs from Figure 1(c) were chosen. In addition, the segment “C3” in Figure 1(a) and the segment “N” in Figure 1(c), which showed particularly sharp dips, were also selected for further analysis. All these regions will be called MoRFs or MoRF regions in the following discussions.

The fragment N of RNase E is located in a structured domain. It has a β-strand at the N-terminus followed by a coil at the C-terminus. A short part of the C-terminal coil has missing electron density in crystal structure [27], but can also form both α-helix [28] and β-strand [27]. Three other segments of interest are located in the intrinsically disordered C-terminal domain of the protein. The RNase E efragment C1 was proposed to be involved in protein self-association [29]. The segment C2 was suggested to interact with structured RNAs and contribute to oligomerization [29]. C3 and C4 regions were observed to bind to enolase and PolyNucleotide Phosphorylase (PNPase), respectively [29].

The MoRF regions of p53 can interact with many other proteins [30]. The following provides a small sampling of a much larger set of experimentally verified. The fragment N of p53 binds to MDM2 [31] and PH-domain of Tfb1 subunit [32]. The C1 segment of p53 can bind to tetrahymena GCN5 [33] and is also responsible for the formation of the p53 tetramer [34]. Finally, the C2 region can interact with four unrelated proteins: cyclin A [35], sirtuin [36], bromodomain of CREB Binding Protein (CBP) [37], and S100ββ[38], and, for these particular examples, there are 3-D structures of the various complexes between the p53 fragment and these four different partners [35–38].

In SRC-3, the N segment is located in the basic Helix-Loop-Helix motif (bHLH). The bHLH motif is well-conserved among other members of the SRC family. Although there are no structural determinations of the of SRC-3 bHLH motif bound with other molecules, this conserved motif of SRC-2 was shown to interact with myogenic factors, such as myogenin and MEF-2C [39], as well as with the Transcriptional Enhancer Factor 4 (TEF-4) [40]. The segments M1 and M2 are so called LxxLL motifs, where L is leucine and x is any amino acid. If the residues designated by x are polar and if the residues before and after this motif are also polar, then this motif would form one turn of an amphiphatic α-helix [41,42] with conserved leucines forming a closely-packed triangle on one face of the helix. This hydrophobic patch can interact with various nuclear receptors [43–46], and are characterized by an adjustable binding affinity [47]. The C1 segment is located in the intrinsic transcriptional Activation Domain 1(AD1) and also contains a LxxLL motif. This segment is responsible for the SRC-3 interaction with general transcriptional co-integrator CBP/p300 [46]. The C2 segment is in the AD2 domain which interacts with Co-Activator-associated aRginine *N*-Methyltransferase 1 (CARM1) and Protein aRginine *N*-MethylTransferase 1 (PRMT1) [48].

Therefore, all the fragments of the three proteins discussed herein are biologically important and are involved in a wide range of specific interactions.

### 2.2. Alignment of MoRFs by Normal Sequential Order

Table 1 lists proteins containing the segments that have similar sequences (with E-value less than 0.0001) to MoRF segments of RNase E, p53, and SRC-3. Apparently, none of the PDB sequences contain segments similar to these MoRF regions under current selection criteria. However, a number of sequences in SwissProt have segments similar to these MoRF regions. In fact, in SwissProt, there are 16 annotated unique proteins and 2 putative proteins. Out of these 16 proteins, 10 are ended with “-ase”, 4 are related to regulation, 1 is related to cell shape, and another one is a chaperone. More interestingly, both hydroxyethylthiazole kinase and rRNA pseudouridylate synthase C have two segments similar to a MoRF of RNase E.

According to our assumption, if these similar segments are flanked by disordered regions, they may have similar binding functions as the original MoRF segments. Hence, PONDR-FIT was applied to predict the disordered status of all these SwissProt sequences.

**Figure 2:**
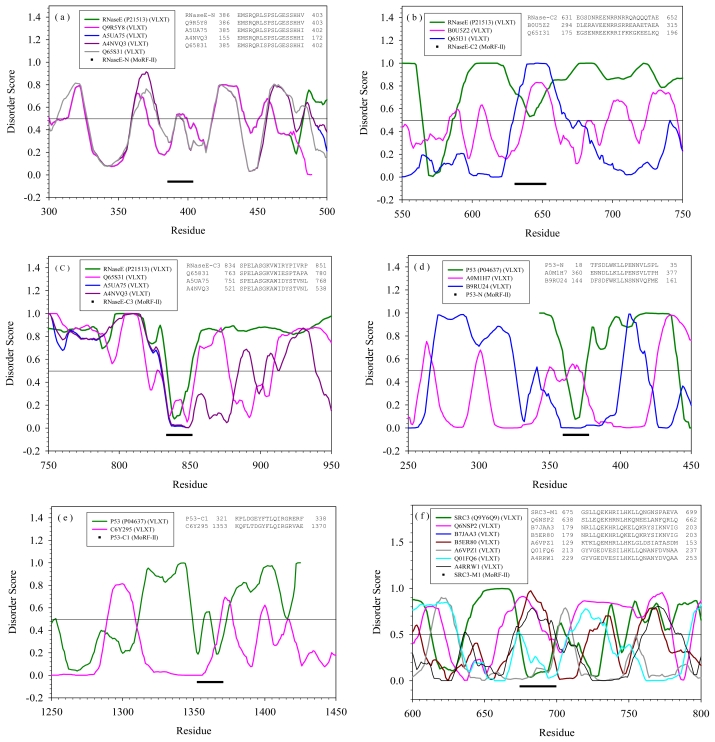

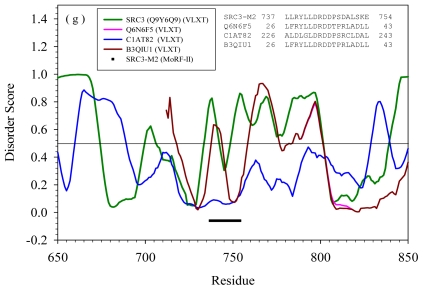
Disorder prediction and sequence alignment for proteins shown in Table 1, which are alignment matches of all the MoRF regions of three proteins in our study. The alignment was cut off at E-value of 0.001. The disorder prediction was implemented by PONDR®VL-XT. The partially sequence alignment is shown as the inset. The insertions in the original alignment were deleted for matching the curve of disorder prediction. The curves of disorder score were shifted to overlap the aligned segments. The N, C2, and C3 MoRF regions of RNase E are shown in (**a**), (**b**), and (**c**), respectively. (**d**) and (**e**) are the N and C1 MoRF regions of P53. (**f**) and (**g**) are the M1 and M2 MoRF regions of SRC-3.

For the purpose of comparing various sequences containing the same MoRF segments, their disorder predictions were presented in the same type of plot (Figure 2). Since seven of the MoRFs listed in Table 1 have sequences similar to unrelated proteins in SwissProt, there are 7 plots in Figure 2 with each plot corresponding to a MoRF segment in Table 1. The inset of each plot is the BLASTP alignment of sequences at the MoRF region. Although there were insertions in the original alignments, these insertions were deleted for the simplicity of matching the curve of disorder prediction.

The MoRF region N of RNase E is highly conserved among five different proteins, where CafA (Q65S31) is also a member of RNase family. As indicated by the prediction in Figure 2(a), this segment in all five molecules is flanked by disordered regions. Although the specific role of MoRF N in RNase E is not clear, the entire N-terminal region of RNase E forms a structured catalytic domain.

Due to this high sequence conservation and matching disordered profile, the segments in four proteins may have functions similar to those of the RNase E MoRF region N. Two different proteins in SwissProt contain segments similar to C2 region of RNase E. However, the peculiarities of order-disorder predictions for these three molecules are quite different. As shown in Figure 2(b), C2 region of RNase E is flanked by disordered regions. The same segment in B0U5Z2 and Q65I31 shows are located in short disordered regions which has only about 25 residues in B0U5Z2 and about 40 residues in Q65I31. Hence, the RNaseE-C2-like segments in both B0U5Z2 and Q65I31 are likely to serve as disordered linkers rather than binding regions.

The C3 region of RNase E was observed to bind to enolase [29]. In STRING 8.2, RNase E was also shown to interact with enolase. As shown by Figure 2(c), Q65S31, A5UA75, and A4NVQ3 all have RNaseE-C3-like segment within a disordered region. The alignment and disorder prediction indicate that these three proteins may also interact with enolase. Actually, as indicated by STRING 8.2, Q65S31 interacts with enolase with high confidence score (~0.999); A5UA75 can also interact with enolase with a score of 0.829. Although the interaction network of A4NVQ3 is missing in STRING 8.2, due to the high sequence identity between A4NVQ3 and Q65S31, it is very likely that A4NVQ3 will also interact with enolase.

The p53-N-like segment was found in two sequences of SwissProt. As illustrated by Figure 2(d), this segment in carbohydrate kinase (A0M1H7) is predicted to be structured in the middle of a long structured domain. The interaction profiles of p53 and A0M1H7 in STRING 8.2 have no common partners. The similar segment in mitochondrial respiratory chain complex assembly protein (B9RU24) seems to contain a MoRF located in a middle of a long loop. It is reasonable that this fragment of B9RU24 could have the same function as the p53-N segment.

The p53-C1-like segment has only one similar sequence in SwissProt, DNA polymerase III alpha subunit (C6Y295). The disordered pattern of this identified protein near the segment is very different from p53. Actually, as indicated by Figure 2(e), this segment is located in a structured region, whereas the C1 segment of p53 is within the disordered region. Hence, it is unlikely that they will have the same binding partner.

The SRC-3-M1 segment is the most commonly matched sequence in SwissProt with six examples, as shown in Figure 2(f). Clearly, the matched segments in Q6NSP2, B5ER80, B7JAA3, and A4RRW1 are predicted to be disordered and are likely to serve as linkers connecting ordered regions. B7JAA3 and B5ER80 segments actually belong to the same protein found in different strains of *Acidithiobacillus ferrooxidans*. In A6VPZ1, the segment is a part of the structured domain. In Q01FQ6, the SRC-3-M1-like segment is located within a disordered region and therefore can potentially serve as a binding motif. Therefore, the only possible candidate having binding partner potentially similar to that of SRC-3-M1 segment is Q01FQ6.

The SRC-M2 segment has three matches in SwissProt. In Figure 2(g), the segment in C1AT82 is a located within a long region predicted to be structured. On the other hand, the segments in Q6N6F5 and B3QIU1 both appear to be structure-prone segments flanked by disordered regions. Besides, the entire sequences of Q6N6F5 and B3QIU1 are almost identical to each other with only several mutations. Thus, these two proteins may also have the same interaction partner as SRC-3.

### 2.3. Reversely Identified Potential Binding Sites

**Figure 3:**
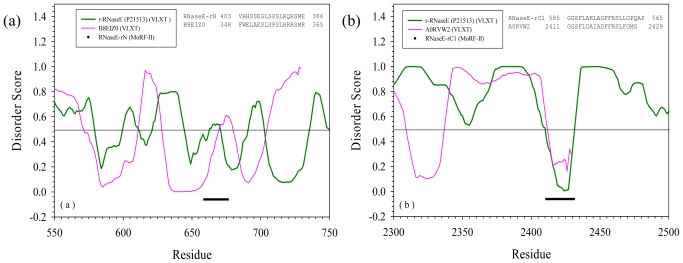

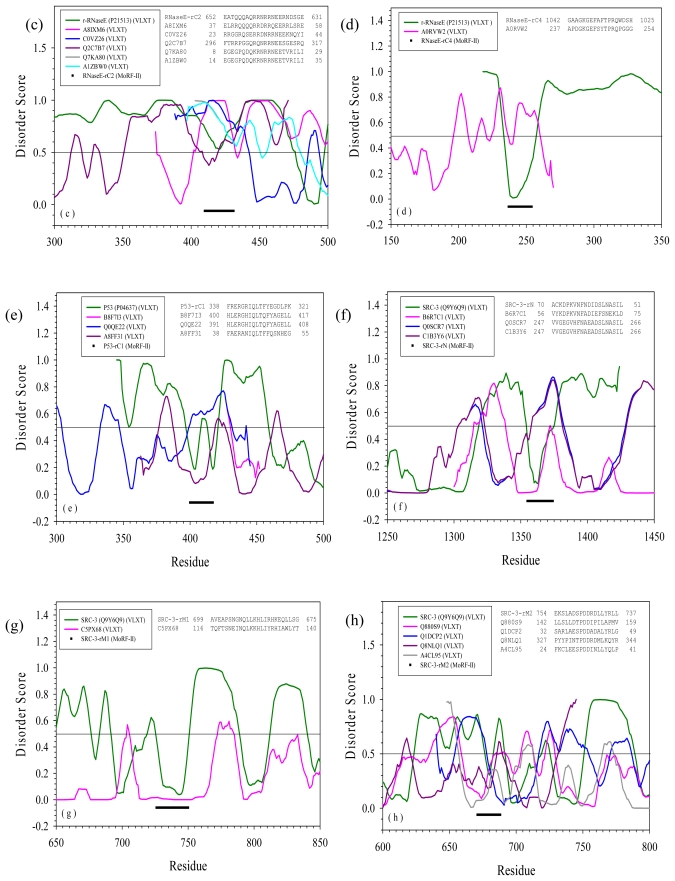

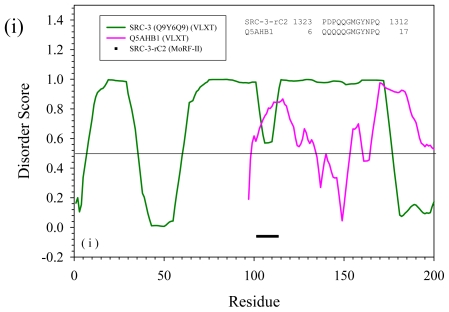
Disorder prediction and sequence alignment for proteins in Table 2, which are reverse alignment matches of all the MoRF regions of three proteins. The alignment was also cut off at E-value of 0.001. Disorder scores were predicted by PONDR®VL-XT. In the insets of sequence alignment, the sequential order of original MoRF regions were shown in a reversed order, while other alignment hits were shown in normal sequential order. The curves of disorder score were also shifted to allowed the overlapped of aligned segments. N, C1, C2, and C4 MoRF regions of RNase E were shown in (**a**)–(**d**), accordingly. C1 MoRF region of P53 was presented in **(e)**. N, M1, M2, and C2 MoRF regions of SRC-3 were plotted in (**e**)–(**i**), respectively.

Table 2 shows sequences containing segments similar to reversed MoRF regions with E-value less than 0.0001. Again, there are no hits in PDB, but quite a few in SwissProt. There are totally 20 proteins in Table 2. The comparison of disorder prediction for proteins containing the same segments is shown in Figure 3. All the sequences in Figure 3 were also shifted to overlap the similar segments. And the original sequences from RNase E, p53, and SRC-3 were inverted to fit the other reversely identified sequences.

The MoRF N segment of RNase E has one reversely aligned match, glycosyl transferase family 2 (B8EIZ0). Figure 3(a) shows that this segment on B8EIZ0 is predicted to be disordered but it is also a connecting segment of two structured regions. Hence, although these two segments highly resemble each other and have almost perfect match of their hydrophobic/hydrophilic patterns, their functional resemblance on binding, if any, is not clear.

The rC1 of RNase E also has one alignment hit in SwissProt, a putative uncharacterized protein (A0RVW2). This protein has 2429 residues. Disorder prediction in Figure 3(b) shows a promising pattern: the RNaseE-rC1-like segment is at the very C-terminal part of A0RVW2 and clearly shows a dip within a disordered region. Both segments have the hydrophilic/hydrophobic pattern expected for an amphipathic helix [41,42]. It is very likely that this RNaseE-rC1-like segment of A0RVW2 will have the similar function as the C1 MoRF region of RNase E. Actually, C1 MoRF region of RNase E is responsible for self-association, and RNaseE-rC1-like segment of A0RVW2 is at the very end of C-terminal, giving some further confidence on its function in self-association.

The rC2 fragment of RNase E has five matches in SwissProt. As indicated by Figure 3(c), the rC2-like segment of A8IXM6 is located in the N-terminal region and is close to a dip within a long disordered region. The similar segment in C0VZ26 is predicted to be highly disordered and is located at the very end of the N-terminus of the protein. The rC2-like segments of Q7KA80, and A1ZBW0 are also located at the N-terminal parts of the corresponding proteins. However, they are predicted to be disordered and contain shallow order dip suggesting that they might be involved in binding.

The similar segment of Q2C7B7 resides close to the C-terminus of the protein and is also predicted to be disordered and contain a shallow order dip. Although there are no obvious conserved hydrophobic sites, these proteins do have interesting patterns of positively and negatively charged residues. This is clearly an indication of their potential ability to bind to DNA and/or RNA. Actually, the C2 region of RNase E is responsible for RNA binding and oligomerization. Therefore, it is quite likely that all the five matched proteins will have the same functions.

The alignment match of rC4 of RNase E in Figure 3(d) shows a similar picture as that for rC1 region of RNase E. The aligned segment of A0RVW2 is located at the C-terminal part of the molecule and shows a dip within the disordered region. This is a sign of a potential binding motif. The sequence pattern may also support the formation of helix. Since the original C4 MoRF of RNase E is responsible for binding to PNPase, it is very likely that A0RVW2 may also bind to PNPase.

In p53, only C1 MoRF region has reversely aligned matches in SwissProt. There are totally three matches as in Figure 3(e). B8F7I3 and Q0QE22 have p53-rC1-like segment at their C-termini. Compared to Q0QE22, B8F7I3 has several mutations. The p53-rC1-like segments of these two proteins are predicted to be disordered. Therefore, it is not clear whether these segments could be involved in binding. As to A8FF31, although the p53-rC1-like segment is close to the N-terminus, it is essentially a part of structured domain. Hence, it is hard to suggest its binding ability.

The MoRF N of SRC-3 has three reverse alignment matches. These segments showed perfect hydrophobic/hydrophilic amphipathic patterns of the helix [41,42]. However, as shown in Figure 3(f), in B6R7C1, the SRC-3-rN-like segment is predicted to be the part of structured domain, whereas similar segments of Q0SCR7 and C1B3Y6 are predicted to be mostly disordered, being located in a disordered linker connecting ordered segments. Hence, by our current criteria, they cannot be identified as possible protein-protein interaction sites. Similarly, the only identified match of the rM1 region of SRC-3 is likely a part of the structured domain (see Figure 3(g)), and therefore, despite the similar helical hydrophobic pattern, the rM1 and the rM1-SRC-3-like fragments are not expected to have the same binding partners.

The M2 region of SRC-3 has four reversed matches in SwissProt. As shown in Figure 3(h), the corresponding segments in Q880S9 and A4CL95 are predicted to be parts of the large structured domains. The reversely-similar segment of Q1DCP2 is located within the N-terminus of the molecule at the beginning of a large structured domain. The identified segment in Q8NLQ1 is also located within the ordered region. Hence, all these segments may not have similar binding function as the original M2 region of SRC-3.

The last segment is the rC2 fragment of SRC-3. It has only one match in SwissProt, Actin cytoskeleton-regulatory complex protein PAN1 (Q5AHB1). As shown in Figure 3(i), the SRC-3-rC2-like segment is at the N-terminal of this 1397-long protein, and is predicted to be fully disordered. Therefore, it may potentially interact with CARM1 and PRMT1 which are the binding partners of the SRC-3 C2 MoRF region.

## 3. Method Section

### 3.1. PONDR-RIBS (Reversely Identified Binding Sites)

The assumption behind PONDR-RIBS is quite straight forward. Suppose a fragment of a protein sequence will form a special non-symmetrical 3D structure and bind to a structured partner. Apparently, the successful binding is decided by not only the structure of the fragment, but also by the structural complementation among all other parts of the protein and the partner. Due to this requirement of structural complementation, reversing the orientation of the fragment may invalidate the binding between the protein and the partner because the structure of the fragment is asymmetric. The integrated structure of the reversed fragment and all other parts of the same protein may not match to the structure of the partner. That is the reason why reverse alignment is not broadly adopted in the research of protein structural biology.

Theoretically, it was expected that a retro-protein; *i.e.*, a protein obtained as a result of reading the sequence backwards, might adapt a topological equivalent of the mirror image of the 3-D structure of its parent protein [1,2,49]. However, the lattice model simulations of the retro-sequence of the B domain of Staphylococcal protein A revealed that the secondary structure elements in the retro-protein did not exactly match their counterparts in the original protein structure [2], and later the full-atom simulation analysis showed that this retro-protein was essentially unfolded [3]. Based on the analyses of inverse sequence similarity in proteins it has been concluded that the tertiary structures of retro-proteins did not imply folds comparable to their parent protein [6]. Furthermore, it was shown that the sequence inversion affected the foldability of some model peptides and proteins in such a way that retro-proteins were generally no more similar to their parent sequences than any random sequence, despite their common hydrophobic/hydrophilic pattern, global amino acid composition and possible tertiary contacts [9]. Therefore, it has been concluded that the direction of protein sequence is a critical factor for the formation of a unique structure. This directionality explains why the sequences of ordered proteins are generally not palindromic [9]. The differences between the parent and retro-proteins likely originate from the differences in the local, detailed structures of amino acids, their dihedral angles, side chain orientations, and packing inside a protein structure.

In agreement with this hypothesis, careful analysis of biologically active retro-protein, retro human metallothionein-2 α domain, revealed that despite the significant alterations in the protein structure induced by the reversal direction of the domain sequence backbone, this retro-domain retained its metal binding ability and foldability mostly due to the fact that reversion of a sequence was not critical to the interaction between Cys side chains and metal ions [9]. Another potential exceptions form the mentioned spatial restrictions are polyproline II (PPII) helices, which tend to occur on the surface of the protein, and PPII-based binding motifs. These structural motifs are left-handed, all-*trans* extended helices with average backbone dihedral angles of (Φ, Ψ) = (−75°, +145°). Each PPII helix has precisely three residues per turn, compared with 3.6 residues per turn in an α-helix. This results in a considerably extended helical structure, with PPII helices translating 3.12 Å per residue compared to 1.50 Å per residue in the α-helix. Each turn of a PPII helix spans approximately 9 Å, resulting in perfect three-fold rotational symmetry [50,51]. Furthermore, residues in PPII helices are significantly more solvent exposed than the average for all residues in ordered proteins, with polar residues in PPII helices 60% more solvent exposed and hydrophobic residues 50% more exposed than the average for all residues [50]. This high surface exposure of both the hydrophobic and polar side chains of residues in PPII conformation provides for an easily accessible hydrophobic or polar interaction surface. As a result, proline-rich sequences are very common recognition sites for protein-protein interaction modules such as the SH3 domain, the WW domain, and the EVH1 domain [52]. For example, the consensus ligand peptides interacting with various SH3-domain-containing proteins in yeast were assigned to class I (RXXPXXP) or class II (PXXPXR) motifs [53], which both regarded as a Pro-rich core LPPLP motif, with the position of the R residue (N or C-terminal to the Pro core) dictating whether the ligand falls in class I or class II [54]. Furthermore, due to the high symmetry, and due to the fact that the PPII helix has three residues per turn, where residues at positions *i* and *i* + 3 lie on the same edge of the ligand structure, PPII-based binding motifs can be inverted. In fact, class I and class II ligands bind to the SH3 domain in reverse orientations relative to each other [55], where a class I ligand binds with its N-terminus at the RT loop and a class II ligand with its C-terminus at this site.

Obviously, the mentioned restrictions in fine structure, dihedral angles, and side chain packing details imposed by backbone directionality that prevent normal folding of retro-sequences can be avoided if intrinsic disorder is taken into account. Intrinsic flexibility of IDPs and IDRs might allow them to gain specific structures needed for successful and specific binding to their partners. Therefore, the spatial hindrance of binding between a reversed fragment and a partner can be conquered by the flexibility of the flanking regions or the binding region itself. Hence, the combination of reverse sequence alignment with disorder analysis might provide very useful information for identifying the possible interaction regions.

Taken fragment **F** in protein **A** can bind to partner **P** and protein **B** contains a segment **rF** which has a reversed sequence of fragment **F**, then the question is whether the protein **B** can interact with partner **P**? To answer this question, the software package, PONDR-RIBS was developed. This new tool provides a synthetic analysis of the binding capability of a reversed fragment and the partners. PONDR-RIBS aligns sequences by CLUSTALW [56] and predicts intrinsic disorder by PONDR-FIT [57]. Here, we restricted the criteria as follows: (1) the sequence identity between reversed fragment **rF** and the original fragment **F** is higher than 60%; (2) The aligned fragment **rF** has similar hydrophobic/charge pattern as that of fragment **F**; and (3) The aligned fragment **rF** is disordered or is flanked by disordered regions. If all these conditions are satisfied, then reversed fragment **rF** of protein **B** might interact with partner **P** with a high probability.

Obviously, the principles of PONDR-RIBS may be used not only for the reversed alignment, but also for the normal-order alignment. Suppose a segment **F** binds to partner **P** and F* is a segment sequentially similar to **F**, the probability of F* binding to **P** should be much higher if F* is flanked by disordered regions or locates in a disordered tail. Hence, by combining the normal-order sequence alignment and disorder prediction, the certainty of identifying binding segments can be significantly improved.

In addition, for proteins having interaction profile in STRING 8.2 [58], we will also cross-reference the results of this database to validate our assumptions on the interaction between two molecules.

### 3.2. Disorder Prediction

Two disorder predictors were applied in this study. The first predictor is PONDR-VLXT [20], which is one of the first disorder predictors. PONDR-VLXT applies various compositional probabilities and hydrophobic measures of amino acids as the input features for the prediction. Although it is no longer the most accurate predictor, it is very sensitive to the local compositional peculiarities of the amino acid sequence. Hence, it is capable of identifying disordered regions possessing increased capability to fold upon interaction with binding partners. Based on this property, another predictor called Molecular Recognition Feature (MoRF) predictor [22,26] was developed to identify the structure-prone segment in a disordered region. The identified segment is known as MoRF region which generally corresponds to the specific dips in the PONDR-VLXT prediction. The second predictor applied in this study is PONDR-FIT [57], which is a meta-predictor combining six individual predictors, PONDR-VLXT [20], VSL2 [59], VL3 [60], FondIndex [61], IUPred [62], and TopIDP [63]. This meta-predictor is a bit more accurate than its individual components and other predictors. Because the identification of possible binding regions relies on the recognition of disordered regions, PONDR-FIT is a good choice for the disorder prediction.

### 3.3. Proteins Studied by PONDR-RIBS

Proteins interact with their partners in an almost endless variability of binding modes. Among them, MoRF is one of the simplest binding motifs which is strongly related to intrinsic disorder [22,26]. MoRFs, short protein segments undergoing disorder to order transition upon binding to a partner, play important functional roles in protein recognition, signaling, and regulation. Often, MoRFs correspond to dips in the PONDR-VLXT plots emphasizing the utility of this computational tool. Although PONDR-VLXT is not the most accurate predictor of intrinsic disorder at the amino acid level, it is absolutely indispensable for finding the short interspersed disordered/structured regions due to its sensitivity to local amino acid composition. These short interspersed disordered/structured regions may have many important types of biological functions. MoRFs, being actually one type of the interspersed structure-prone motif within disordered regions, are highly abundant in protein sequences. In fact, over 40% proteins in eukaryotes genomes are predicted to contain at least one α-helical MoRF [22,64].

Due to these considerations, three disordered proteins containing multiple MoRF regions were selected for further study, *i.e*., RiboNuclease E (RNase E) (SwissProt id: P21513), p53 (SwissProt id: P04637), and Steroid Receptor Co-activator 3 (SRC-3) (Swissprot id: Q9Y6Q9). RNase E is an important enzyme in the pathway of mRNA degradation. p53 plays a number of important roles in cell differentiation, development, and genome stability. SRC-3 assists the regulation of gene expression. Each of these three proteins binds to many partners through their MoRF regions. By our assumption, a query protein containing fragment similar to these MoRFs or their reversed versions may also interact with the same partners.

### 3.4. Alignment against Various Protein Databases

PONDR-RIBS provides synthetic comparison between a segment and protein sequences by applying multiple sequence alignments and disorder prediction. However, these two steps are extremely rate-limiting in the whole process. In addition, multiple sequence alignments on a large number of proteins may produce a very complicated pattern of insertions and deletions, thus increasing the difficulty of analysis. In this paper, to improve the efficiency of identifying of the possible identical segments in a database, BLASTP was used and all sequences with E value less than 0.001 were pre-selected as the potential candidates. After this pre-selection step, PONDR-RIBS was applied for further analysis.

## 4. Conclusions

Two well-accepted bioinformatics tools, sequence alignment and disorder prediction, were combined to probe possible binding partners in protein databases. For MoRF regions [22,26], it is clear that such combination has many advantages in identifying the possible binding interactions. Furthermore, based on the rationalization of structural properties of disordered proteins, a method called reverse alignment was also proposed to identify the potential interactions between the reversely-similar segment and the partners of the original fragment.

In this paper, when applying BLAST to search similar segments in PDB and SwissProt, a small E-value of 0.0001 is applied. The results from small E-value have many advantages: a limited number of examples with high confidence; high efficiency in analyzing the biologically important functions of these example proteins; simplicity in explaining the rules of application. However, important examples may be overlooked. In this newly developed method, the general intrinsic disorder and the hydrophobic pattern are more important than the value of confidence. Actually, in the case of segment N of RNase E, increasing E-value from 0.0001 to 0.1 resulted in finding two additional possible binding partners. These additional possible partners also had sound conserved hydrophobic patterns and interesting disorder prediction. Further increase of the E-value to 1000 produced more than 70 proteins. All these proteins had more than 50% sequence identity to the segment and partially kept the hydrophobic pattern. However, the increased E-value provided more candidates and increased the technical difficulty of analysis. Therefore, in this paper, the attention was focused at lower E-values and small amount of candidates.

The comparison of species in Tables 1 and 2 is very interesting. In our study, the sequence of RNase E is from *E. coli*, while other three sequences (p53, SRC-3, and 4E-BP1) are all from human. As shown in Table 1, the N MoRF of RNase E has three matches, a fragment of hydroxyethylthiazole kinase (A5UA75), rRNA pseudouridylate synthase C (A4NVQ3), and CafA proteins (Q65S31). The first two proteins are from *Haemophilus influenza*, while the last one is from *Mannheimia succiniciproducens*. Although more solid evidence is required, the possibility of trans-species gene transfer and the functional conservation of transferred gene are very interesting. Furthermore, both proteins from *Haemophilus influenza* have two segments similar to the corresponding segments in RNase E of *E. coli*. Further analysis shows that there is a high level of sequence conservation between these two proteins. In comparison with hydroxyethylthiazole kinase, rRNA pseudouridylate synthase C has an extra C-terminal region. These two proteins may share the same reading frame.

As indicated by the comparison of the interaction profiles of RNase E, A5UA75, and Q65S31 in STRING 8.2 [58], with high confidence these three proteins can interact with PNPase, Enolase, Protein hfq, and 60 kDa chaperonin. It is known that C3 and C4 MoRF region are responsible for the binding to Enolase and PNPase, respectively [29]. However, A5UA75 and Q65S31 do not have the segments similar to the C4 region of RNase E. Therefore, there is still a question about the existence of common regions of binding to PNPase among these three proteins. Besides this, the common regions interacting with Protein hfq and 60 kDa chaperonin are also unknown. This is also the indication that more deliberate techniques are required in future study.

The normal-order sequence alignment results in 12 possible candidates out of 21 for 13 segments of three proteins as in Table 1, while the reversely identified sequences in Table 2 are 10 out of 20. As indicated by these data, the combination of sequence alignment and disorder prediction may greatly narrow down the number of high-confidence interaction partners. Furthermore, the reverse alignment may discover new interaction partners as effective as the normal-order alignment. This is definitely important for our further understanding of protein-protein interaction networks.

## Figures and Tables

**Table 1:** MoRFs of three proteins and their alignment matches in PDB and SwissProt.

Protein	MoRF	Proteins in PDB containing similar MoRF (a)	Proteins in SwissProt containing similar MoRF (a)			
			SwissProt id	Species	Name	Within IDR
RNase E	N	---	Q9R5Y8	*E. Coli*	Cell shape determining protein	Yes
			A5UA75	Haemophilus influenzae	Hydroxyethylthiazole kinase	Yes
			A4NVQ3	Haemophilus influenzae	rRNA pseudouridylate synthase C	Yes
			Q65S31	Mannheimia succiniciproducens	CafA protein	Yes
	C1	---	---		---	---
	C2	---	B0U5Z2	Xylella fastidiosa	Glutamyl-tRNA reductase	Yes
			Q65I31	Bacillus licheniformis	Anthranilate synthaseTrpE	Yes
	C3	---	A5UA75	Haemophilus influenzae	Hydroxyethylthiazole kinase	Yes
			A4NVQ3	Haemophilus influenzae	rRNA pseudouridylate synthase C	No
			Q65S31	Mannheimia succiniciproducens	CafA protein	No
	C4	---	---		---	---
p53	N	---	A0M1H7	Gramella forsetii	Carbohydrate kinase	Yes
		---	B9RU24	Ricinus communis	Mitochondrial respiratory chain complexes assembly protein, putative	
	C1	---	C6Y295	Pedobacter heparinus	DNA polymerase III, α subunit	No/Yes
	C2	---	---		---	---
SRC-3	N	---	---		---	---
	M1	---	Q6NSP2	Zebrafish	Rho/rac guanine nucleotide exchange factor (GEF)	Yes
			B7JAA3	Acidithiobacillus ferrooxidans	Nif-specific regulatory protein	Yes
			B5ER80	Acidithiobacillus ferrooxidans	Transcriptional regulator, NifA, Fis Family	Yes
			A6VPZ1	Actinobacillus succinogenes	Sulfite reductase [NADPH] hemoprotein beta-component	No
			Q01FQ6	Ostreococcus tauri	CLP protease regulatory subunit CLPX (ISS)	Yes
			A4RRW1	Ostreococcus lucimarinus	Mitochondrial ClpX chaperone	Yes
	M2	---	Q6N6F5	Rhodopseudomo nas palustris	ATP-dependent DNA helicase	Yes
			C1AT82	Rhodococcus opacus	Hypothetical membrane protein	No
			B3QIU1	Rhodopseudomonas palustris	DEAD/DEAH box helicase domain protein	Yes
	C1	---	---		---	---
	C2	---	---		---	---

(a) Only proteins different from the original protein and its family are listed.

**Table 2:** Reversed segments of MoRFs of three proteins and their alignment matches in PDB and SwissProt.

Protein	MoRF(a)	Proteins in PDB containing similar MoRF (b)	Proteins in SwissProt containing similar MoRF (b)			
			SwissProt id	Species	Name	Within IDR
RNase E	rN	---	B8EIZ0	Methylocella silvestris	Glycosyl transferase family 2	Yes
	rC1	---	A0RVW2	Cenarchaeum symbiosum	Putative uncharacterized protein	Yes
	rC2	---	A8IXM6	Chlamydomonas reinhardtii	Dopamine beta-monooxygerase-like protein	Yes
			C0VZ26	Actinomyces coleocanis	30S ribosomal protein S5	Yes
			Q2C7B7	Photobacterium	Pseudouridine synthase	Yes
			Q7KA80	Drosophila melanogaster	Heterogeneous nuclear ribonucleoprotein	Yes
			A1ZBW0	Drosophila melanogaster	Bancal isoform C	Yes
	rC3	---	---		---	---
	rC4	---	B9LNU7	Halorubrum lacusprofundi	Manganese containing catelase	Yes
p53	rN	---	---		---	---
	rC1	---	B8F7I3	Haemophilus parasuis serovar 5	tRNA modification GTPase TrmE	Yes
			Q0QE22	Haemophilus parasuis	ThdF	Yes
			A8FF31	Bacillus pumilus	3-dehydroquinate dehydratase	No
	rC2	---	---		---	---
SRC-3	rN	---	B6R7C1	Pseudovibrio	Outer surface protein	No
			Q0SCR7	Rhodococcus	Aldehyde dehydrogenase	Yes
			C1B3Y6	Rhodococcus opacus	Phenylacetic acid degradation protein PaaN	Yes
	rM1	---	C5PX68	Sphingobacterium spiritivorum	Conserved hypothetical transmembrane protein	No
	rM2	---	Q880S9	Pseudomonas syringae	AraC-family transcriptional regulator	No
			Q1DCP2	Myxococcus xanthus	Tetratricopeptide repeat protein	No/Yes
			Q8NLQ1	Corynebacterium glutamicum	UDP-galactopyranose mutase	No
			A4CL95	Robiginitalea biformata	Type III restriction enzyme	No
	rC1	---	---		---	---
	rC2	---	Q5AHB1	Candida albicans	Actin cytoskeleton-regulatory complex protein PAN1	Yes

(a) “r” stands for reversed segment.

(b) Only proteins other than original proteins and its family are included.
